# Open area segmentation in CT images based on pixel displacement and multi-view with application in the axillary and lower cervical regions

**DOI:** 10.1002/mp.70210

**Published:** 2025-12

**Authors:** Tiange Liu, Yajie Wang, Drew A. Torigian, Yubing Tong, Zhaochen Yang, Jayaram K. Udupa

**Affiliations:** 1 School of Intelligence Science and Technology, University of Science and Technology Beijing, Beijing, China; 2 School of Information Science and Engineering, Yanshan University, Qinhuangdao, Hebei, China; 3 Medical Image Processing Group, Department of Radiology, University of Pennsylvania, Philadelphia, Pennsylvania, USA; 4 Department of Computer Engineering, Tandon School of Engineering, New York University, Brooklyn, New York, USA

**Keywords:** axillary region, lower cervical region, medical image segmentation, pixel displacement

## Abstract

**Background:**

In medical image segmentation, the segmentation of open regions presents challenges and remains relatively unexplored. Unlike objects such as organs that have well-defined boundaries, open regions typically lack clear boundaries, complicating the segmentation process.

**Purpose:**

This study aims to propose a new segmentation algorithm to address these challenges in the segmentation of open regions and to improve the accuracy and reliability of segmentation.

**Approach:**

This paper introduces a segmentation algorithm inspired by the concept of optical flow, which leverages pixel displacement information within a 2.5D CNN framework. Our framework fully exploits the potential of multi-view networks, achieving precise segmentation of open areas in axillary and lower cervical regions by integrating information from multiple views and pixel tracking capabilities.

**Results:**

We validated the proposed algorithm using two CT datasets from the axillary region and the lower cervical region. This dataset includes a total of 100 patient studies, which are randomly selected and allocated, with 50 allocated for training, 10 for validation, and 40 for testing purposes. Experimental results show that the proposed algorithm achieves Dice coefficients of 90.25% and 80.43% in these two regions, surpassing the state-of -the-art methods by over 1.32% and 1.85%, respectively. The ablation experiments confirm that both the multi-channel ensemble and pixel displacement guidance strategies enhance segmentation accuracy in open regions.

**Conclusions:**

The algorithm captures the dynamic changes occurring in the region by analyzing the intensity changes information of pixels between adjacent slices, effectively addressing the segmentation challenges of open region boundaries. This is particularly evident in areas with step-like changes, where the algorithm incorporates pixel displacement information as prior knowledge along with a multi-channel ensemble strategy to enhance segmentation accuracy and robustness.

## 1 | INTRODUCTION

The human body consists of various types of tissue, each of which plays a vital role in sustaining the structure and function of the body.^1^ These tissues do not operate independently but are instead interconnected with surrounding tissues to form complex areas, such as the axillary region and the lower cervical region. These regions contain substantial amounts of adipose tissue and clusters of lymph nodes, as well as important nerves, arteries, veins, and some small muscles. These areas are characterized by hints of intensity boundaries along with often large discontinuities or gaps in the boundary where no boundaries are observable. As such, the hinted boundary segments constitute topologically non-closed surfaces in three-dimensional (3D) space.^2^ Yet, these areas constitute meaningful 3D objects once a conceptual anatomic definition is formulated. We name these objects as “open regions.” This term reflects the fact that these objects comprise multiple types of tissue and possess indistinct or incomplete boundaries, which makes them challenging to delineate accurately, as depicted in Figure 1.

The axillary region, which encompasses the left and right axillary regions together, is delineated as a pyramidal “virtual space” or a conceptual object that is characterized by a substantial presence of adipose tissue, axillary lymph nodes, and vital neurovascular structures. This anatomical area is of paramount importance for the functional integrity of the upper limb. Specifically, the axillary artery serves as the principal source of blood supply to the upper limb, facilitating perfusion to the shoulder, chest, and upper arm. Concurrently, branches of the brachial plexus are responsible for providing nerve innervation to the axilla, shoulder, and upper limb, thereby ensuring the maintenance of sensory and motor function.^3,4^ The lower cervical region, which encompasses the left and right lower cervical regions together (including the left and right supraclavicular regions), is bordered by the skeletal muscles of the neck, upper chest, and upper limbs, and encompasses critical components such as lower cervical and supraclavicular lymph nodes, nerves, and blood vessels. This region is crucial for facilitating neck mobility, ensuring head stability, and enabling effective nerve conduction. These open regions not only contain critical anatomical structures but also serve as potential sites for a variety of pathological conditions such as lymphadenopathy (i.e., the presence of abnormal lymph nodes due to spread of cancer or other pathologies). As such, accurate auto segmentation of these regions in medical images may play a major role in diagnostic radiology to facilitate disease detection, quantification, staging, and response assessment, as well as in radiation oncology for the purpose of treatment planning.^5–14^

Currently, proven methodologies for segmentation and subsequent quantitative analysis of open regions such as these are lacking.^15–20^ Therefore, this research aims to achieve effective segmentation of these open regions. Table 1 presents brief anatomic definitions of the two open regions of interest in this study such that precise manual ground truth segmentations can be created for use in building appropriate segmentation models. Our definition documents provide detailed contouring instructions with examples illustrating tracings on a range of slices going from the superior-most slice to the inferior-most slice of each open region.

In recent years, the rapid advancement of deep learning technologies, particularly the remarkable efficacy of convolutional neural networks (CNNs) in natural and medical image segmentation, has facilitated increasingly precise segmentation of target objects. Among these developments, U-Net^21^ emerges as a prominent architecture, adeptly integrating low-level and high-level features through the implementation of skip connections, thereby achieving high-precision segmentation outcomes. To further enhance segmentation performance, various improved iterations have been proposed, including UNet++^22^ and ResUNet++^23^, which augment segmentation accuracy through distinct connection methodologies and advanced feature enhancement techniques.

Notwithstanding the considerable strides made by diverse CNN architectures in the realm of medical image segmentation, research specifically addressing boundary-related challenges remains insufficiently explored. Several studies have concentrated on the restoration of boundary details by reinforcing pixel dependencies.^24,25^ Additionally, interactive segmentation methods have incorporated user inputs, such as points and bounding boxes, into segmentation networks.^26,27^ While these studies mainly address the segmentation difficulties arising from ambiguous boundaries and heterogeneous textures attributed to contrast variations, a comprehensive investigation into segmentation of open regions has yet to be conducted.

Mo et al.^28^ developed a substantial dataset of image-displacement patch pairs, utilizing image masks to facilitate the learning of displacements for boundary tracking in target regions. Their methodology was applied to heart segmentation within the x-ray chest SCR dataset, characterized by visually non-closed boundaries. However, this approach is constrained to images containing a single target region, presenting challenges when attempting to segment multiple targets simultaneously. Moreover, it necessitates an initial point that is relatively in proximity to the target region to ensure the accuracy and effectiveness of segmentation.

This paper proposes a novel 2.5D-based, pixel displacement-guided automatic segmentation framework, specifically targeting the axillary and lower cervical regions. The objective is to effectively address the segmentation of these two open regions in CT images. The contributions of this study are as follows:

1. We innovatively leverage pixel displacement information between adjacent slices to confront the segmentation challenges associated with open regions. By quantifying the movement information of pixels between successive slices and integrating this as prior knowledge into the segmentation network, we enhance our ability to accurately capture spatial changes in the target area across slices, facilitating the segmentation of images with multiple target regions without reliance on the selection of an initial point or other forms of interactive user input.
2. We introduce a multi-channel ensemble strategy designed to address the step-like changes in the segmentation of open regions. While pixel displacement is proficient in tracking boundary transitions, its performance can be impaired in cases of abrupt changes in the target region and proves challenging in 3D imaging contexts. By implementing a multichannel ensemble strategy, we effectively overcome these limitations, resulting in improved segmentation accuracy.

### 1.1 | Related work

In the domain of medical image segmentation, segmentation models are primarily classified into two categories: 2D and 3D methods. The 2D approaches are frequently preferred due to their superior computational efficiency and inference speed. Nevertheless, these methods typically process images in a sequential manner, layer by layer, and in a specified direction, which restricts their ability to capture critical contextual information between adjacent slices. This limitation hampers the effective utilization of implicit relationships present among neighboring slices during the segmentation of anatomical structures, potentially resulting in inaccuracies such as structural breaks or discontinuities.

Conversely, 3D segmentation methods are capable of comprehensively considering the spatial information of anatomical structures, thereby offering deeper insights into the interrelationships between different layers. Architectures such as 3D U-Net^29^ and V-Net^30^ are designed to capture volumetric features through the application of 3D convolutions. These architectures enhance feature learning by incorporating residual connections and attention mechanisms, thus facilitating more precise segmentation of intricate anatomical structures. However, 3D models necessitate considerable computational resources, which results in extended training times and complicates the implementation process. Consequently, such factors restrict the applicability of 3D models in environments with limited resources. Additionally, 3D models are susceptible to overfitting when applied to small datasets, compromising their generalization capabilities.

In this context, the 2.5D approach represents a balanced solution, effectively reconciling computational efficiency with information capture capabilities, thus rendering it a viable option. The 2.5D medical image segmentation approach seeks to utilize structural features while minimizing computational expenses. These methods can be broadly categorized into three types.^31^ The first category involves the integration of 2D segmentation outcomes obtained from various views, including sagittal, coronal, and axial planes.^32,33^ The second category emphasizes leveraging the relationships between slices to consolidate slice features.^34,35^ The third category entails the concurrent training of both 2D and 3D segmentation networks, followed by the fusion of their features.^36–38^ In this study, only the first two categories are considered for utilization to build our method.

#### 1.1.1 | Multi-view ensemble

Due to the characteristics of the imaging, the target may exhibit pronounced step-like variations from slice to slice. Acquiring information from multiple perspectives at different scales can effectively mitigate the effects of these step-like changes. Consequently, a multi-view ensemble strategy is particularly well-suited for our task.

In recent years, the application of multi-view ensemble techniques for 3D medical image segmentation has advanced. Some studies have sliced 3D data into coronal, sagittal, and axial views, converting them into a format that is compatible with 2D fully convolutional networks.^39,40^ While this approach is intuitive and effective, it has a drawback: it cannot adequately capture the continuity of structures in the same direction, which may result in the loss of critical contextual information. Solely relying on multi-view ensemble methods does not sufficiently track relevant information between adjacent slices within a single view. This limitation can reduce the accuracy of medical image segmentation, particularly when dealing with complex anatomical structures.

Regarding fusion methods, there are several challenges beyond the commonly used majority voting. Majority voting neglects the proportion of segmentation results from different directions and fails to fully utilize the information available from each perspective, thus providing an insufficient basis for decision-making to enhance overall segmentation performance. Xia et al.^41^ proposed a 3D Volume Fusion Network to integrate results from 2D networks trained from various perspectives. However, it is important to recognize that the computational cost of training 3D networks remains relatively high.

#### 1.1.2 | Information tracking from slice to slice

To effectively leverage the spatial information inherent in volumetric data, researchers have explored the direct and indirect transmission of information between slices.^42–48^ For the direct category,^42–44^ they involve the extraction of multiple 2D slices, followed by the application of a sliding window approach to integrate consecutive slices into multi-channel 2D images, which serve as input for 2D models. Although these methods capture relationships between adjacent slices at a lower computational cost, directly stacking neighboring slices into multi-channel 2D images fails to model complex context and dynamic changes, limiting their ability to fully exploit these relationships and potentially leading to inaccuracies in segmentation. Some studies from the indirect category have sought to enhance contextual information between slices for 3D segmentation by integrating CNNs with recurrent neural networks,^45–48^ thereby modeling the dependencies and contextual relationships between slices, demonstrating noticeable improvements in the performance of CT image segmentation. However, despite these advancements, such methodologies still exhibit limitations in monitoring boundary changes in open regions.

This approach is extensively applied within the realm of video analysis and can be categorized into two types based on training methods: supervised and unsupervised. Supervised optical flow estimation learns features and motion patterns from large labeled optical flow datasets, employing classical supervised deep learning models, including FlowNet^49^ and PWC-Net.^50^ FlowNet marked the inaugural effort to predict optical flow directly using CNNs. Prior to the advent of FlowNet, optical flow prediction predominantly relied on traditional methodologies such as the variational Horn–Schunck method^51^ and the Lucas–Kanade method.^52^ FlowNet utilizes an encoder–decoder architecture to extract features at various scales, capturing both details and contextual information regarding optical flow, thereby framing the optical flow prediction problem as a supervised deep learning task. PWC-Net features a pyramid-structured design that leverages pyramid features to enhance the precision and stability of optical flow, introducing a flow context network to bolster flow consistency. Although supervised training methods typically yield high accuracy and effectiveness, their performance may diminish when optical flow data are scarce, thereby constraining their applicability in real-world contexts.

To mitigate dependency on labeled data, researchers have proposed unsupervised optical flow estimation methods. These techniques harness deep learning frameworks to derive optical flow from unlabeled image sequences, utilizing deep neural networks for image feature extraction and representation learning. In contrast to supervised methods, unsupervised optical flow estimation does not necessitate manually labeled data. It relies instead on prior assumptions regarding the self-consistency of image sequences and motion smoothness during training, enhancing scalability and practicality. Nonetheless, since unsupervised optical flow estimation does not depend on training data with optical flow annotations and lacks explicit supervisory signals for model learning, its performance may decline in complex scenarios within input image sequences, such as instances of data loss, noise, occlusions, and blurring of motion boundaries. To address these challenges, UnFlow^53^ utilizes unsupervised census transforms to account for occlusion conditions affecting bidirectional optical flow values. EpiFlow^54^ employs low-order constraints in a subspace alongside global kernel geometric constraints to alleviate challenges posed by repetitive textures and occluded regions. Inspired by patch matching methods, PatchFlow^55^ introduces a parallel decoder branch to learn occlusion masks, implementing bidirectional consistency checks to derive the loss function for occlusion masks. Although the aforementioned studies contribute to mitigating occlusion-related issues, they still necessitate a substantial amount of high-quality unlabeled frames for training to achieve optimal performance, which may be challenging to obtain in certain circumstances.

While optical flow-based methods adeptly capture continuous local information from successive frames via the optical flow field, thereby effectively tracking the movement trajectory of the target boundary, they exhibit limitations in addressing abrupt changes. Optical flow algorithms generally presume smooth motion of the target between consecutive frames. In regions characterized by considerable variations, the optical flow field often fails to deliver precise motion estimates. Consequently, in areas such as the lower cervical region, where sudden alterations occur in the shape or position of the target, the predictive accuracy of optical flow methodologies markedly decline.

## 2 | METHODS AND MATERIALS

The workflow in our approach is illustrated in Figure 2. We also provide a concise pseudocode of our method in Table S1.

To begin, we slice the 3D images into 2D sections along the axial, sagittal, and coronal planes, which are then processed by the same 2D network. Figure 2 demonstrates the training scenario using axial slices. Subsequently, we combine the intermediate slices with their neighboring counterparts to form multi-channel inputs. This technique enables us to extract key features not only from the individual slices but also from adjacent ones. The displacement of pixels between consecutive frames acts as crucial motion information and provides the network with prior knowledge. The pixel displacement visualization results are shown in Figure S1. In the next phase, we utilize pixel displacement information as a guiding cue to assist the network in tracking boundaries. This motion-based cue enhances the network’s capability to focus on and accurately delineate the boundaries of open regions throughout the segmentation process. Finally, we implement a weighted voting fusion of the segmentation results from the slices at the three orientations to incorporate anatomical structural features. This method effectively addresses the challenges associated with open region segmentation. In this section, we will elaborate on the framework guided by neighboring slice information for open region segmentation, along with the rationale behind our approach.

### 2.1 | Multi-channel ensemble learning

In medical image analysis, traditional 2D CNNs mainly focus on information within axial (transverse) plane images, often overlooking spatial information from other directions. This limitation can result in fragmented or incomplete segmentation outcomes in 3D space. A multi-view ensemble strategy is regarded as an effective solution, as it integrates spatial information from various perspectives.

As shown in Figure 3, slices obtained from different directions exhibit notable differences in shape and size. In the sagittal plane (second row), the axillary region shows notable differences in size and shape compared to the other two planes, with fewer open regions present. This indicates clearer boundary delineation in this direction, which provides additional information for the other two planes. In contrast, both the axial plane (first row) and coronal plane (third row) demonstrate similarities in size and shape. However, the variations in open regions suggest that the model emphasizes different open areas in these planes, leading to distinct segmentation challenges. By integrating the learning outcomes from these planes, we can effectively enhance the segmentation accuracy of open regions.

To fully utilize the information from different planes, we propose a multi-channel ensemble approach. By introducing a multi-channel mechanism, the model can capture the continuity of features between slices in each direction, thereby recording more detailed variations. We stack consecutive 2D medical image slices to create input channels for the model, with each channel representing a slice sized H x W. The number of stacked slices, C, determines the number of input channels. Instead of processing the entire 3D volume directly, we approximate the 3D spatial information by stacking adjacent 2D slices. We set C = 2s + 1, where s denotes the number of continuous 2D image slices stacked around the center slice. For slices lacking adjacent top or bottom slices, we utilize the image slice itself as its neighboring slice.

We input multi-channel images from different planes into three identical networks for training to gather structural information from various directions. Finally, based on the segmentation results and characteristics of each plane, we assign different weights to each result and perform a weighted summation to obtain the final segmentation outcome. This process helps compensate for the limitations of a single model, as shown in Equation (1).

(1) $$R_{\text{fin}}=w_{t}\cdot R_{t}+w_{s}\cdot R_{s}+w_{c}\cdot R_{c},$$

where *R*_t_, *R*_s_, and *R*_c_ represent the segmentation results of the axial, sagittal, and coronal planes, respectively, while *w*_t_, *w*_s_, and *w*_c_ represent the corresponding weights that reflect the significance of the results from each plane. *R*_fin_ denotes the final segmentation result.

### 2.2 | Pixel tracking by pixel displacement information

In contiguous slices, there are subtle differences in the open regions between adjacent slices. Whether these differences expand outward or contract inward,the magnitude of the changes is typically minimal. We leverage this shape and appearance contiguity characteristic to extract contextual information from the slices, guiding the network to concentrate on pixel variations that address the segmentation challenge in open regions. Specifically, we introduce an additional branch to a two-dimensional network built on an encoder–decoder architecture, where this branch shares backbone features with the decoder. By incorporating pixel displacement information between adjacent slices, we enhance the model’s capacity to track shape changes in the target region.

In contrast to conventional two-dimensional networks that depend solely on backbone features for segmentation predictions, we utilize the original prediction results as supplementary cues to create segmentations that integrate information from adjacent slices. To achieve segmentation of the current slice $S_{n}$, we first calculate the pixel displacement distance between the embedding feature $f_{n}^{\text{emb}}$ of the current slice and the embedding feature $f_{n-l}^{\text{emb}}$ of the adjacent slice $S_{n-l}$, thereby obtaining the distance map $D_{n}$, which will be explained in detail in Sec. 2.2.1 and Sec. 2.2.2.

Next, the distance map $D_{n}$ is concatenated with the original prediction $P_{n-l}^{\text{ori}}$ of the adjacent slice $S_{n-l}$, and the backbone features $f_{n}$ of the current slice to generate the refined result $P_{n}^{\text{ref}}$ for the current slice, as indicated in Equation (2).

(2) $$P_{n}^{\text{ref}}=conv\;\left(D_{n}⊕P_{n-l}^{\text{ori}}⊕f_{n}\right),$$

here, ⊕ refer to the concatenation operation, and “conv” signifies a convolutional layer that is made up of a batch normalization process and a rectified linear unit (ReLU) activation layer. Finally, we implement a squeeze-and-excitation attention mechanism to adjust the weights of each channel, thereby enhancing the focus on important channels to achieve the final segmentation result $P_{n}^{\text{fin}}$ of the current slice, as shown in Equation (3).

(3) $$P_{n}^{\text{fin}}=SE\;\left(P_{n}^{\text{ref}},P_{n}^{\text{ori}}\right),$$

where $P_{n}^{\text{ori}}$ represents the original prediction of the current slice. Throughout this process, we use the pixel movement information between adjacent slices as cues to guide the network during end-to-end training.

#### 2.2.1 | Embedding space

Convolution blocks are utilized to derive the embedding features $f_{n}^{\text{emb}}$, the convolutional block consisting of a 3 × 3 convolutional layer, a batch normalization layer, and a ReLU activation layer. This procedure non-linearly transforms high-dimensional features into a lower-dimensional space, enhancing the intuitiveness of the relationships and characteristics within the features.

To obtain the embedding representation $f_{n-l}^{\text{emb}}$ of adjacent slices, we cyclically shift the backbone embedding features of the current slice along the batch dimension where we treat the depth dimension as the batch dimension in this implementation, as illustrated in Figure 4. In this context, *n-l* refers to the previous *l* slices. For scenarios with less pronounced stepping, a larger value for *l* can be selected to capture a more extensive spatial context. However, for the two regions examined in this study—particularly the lower cervical region—there is a marked stepping characteristic. Consequently, we set *l* to 1, which corresponds to the backbone embedding features of the immediately preceding slice.

#### 2.2.2 | Pixel displacement calculation

When predicting the current slice, we calculate the pixel displacement distance by comparing the embedding feature $f_{n}^{\text{emb}}$ of the current slice and the embedding feature $f_{n-1}^{\text{emb}}$ of the adjacent slice. This allows us to capture subtle shape variations within the image sequence. To represent pixel displacement distances, we assess the degree of matching between image patches in $f_{n}^{\text{emb}}$ and $f_{n-1}^{\text{emb}}$. The detailed calculations are illustrated in Figure 5.

The rectangular block $e_{x_{1}}$ centered at pixel $x_{1}$ in $f_{n-1}^{\text{emb}}$ and the rectangular block $e_{x_{2}}$ centered at pixel $x_{2}$ in $f_{n}^{\text{emb}}$, both with a search radius *k* for correlation matching, are analyzed. Specifically, unlike FlowNet which uses dot product to compute matching scores, we calculate the distance between *e*_*x*_1__ and *e*_*x*_2__ using the Euclidean distance to enhance the interpretability of spatial differences. We then normalize the distance using Equation (4) to obtain the matching distance value for pixel *x*_1_.

(4) $$d\left(x_{1},x_{2}\right)=1-\frac{2}{exp\left({\left\|e_{x_{1}}-e_{x_{2}}\right\|}^{2}\right)+1},$$

By computing the matching degree between the block associated with pixel $x_{1}$ in $f_{n-1}^{\text{emb}}$ and all the blocks associated with pixels in $f_{n}^{\text{emb}}$, we can infer the potential displacement of pixel *x*_1_ . Subsequently, using Equation (5), we select the block with the highest matching degree as the final movement distance for pixel *x*_1_.

(5) $$D_{n}\;\left(x_{1}\right)=\underset{x_{2}\in P_{n-1}}{min}d\left(x_{1},x_{2}\right),$$

Here,the matching distance $d\left(x_{1},x_{2}\right)$ ranges from 0 to 1. A higher matching degree corresponds to a $d\left(x_{1},x_{2}\right)$ value closer to 0, indicating proximity between the pixels, while a lower matching degree results in a value closer to 1, signifying greater distance. We calculate the correlation matching degree between all pixels and generate the distance map.

Given that calculating the distance between all pixels in the two feature maps can be computationally intensive, we exploit the observation that pixel displacement in medical images is generally constrained within a fixed range. During the correlation calculation, we utilize a fixed-size search region and disregard pixels outside this boundary. Furthermore, we take advantage of the original prediction results from the adjacent slice to limit the computational range, focusing solely on pixel displacements within the prediction *P*_*n*−1_.

Different from the optical flow-based method, the matching quality in our method is assessed by computing the Euclidean distance between embedded feature vectors, yielding a spatial distance map that reflects tissue deformation. Besides, instead of using displacement as the final output, we employ it as prior knowledge to guide the segmentation process. While maintaining computational efficiency, our method demonstrates superior accuracy compared to optical flow approaches, particularly in segmentation tasks of the lower cervical region, as detailed in Table 3 of Section 3.

#### 2.2.3 | Feature fusion

We fuse the refined result *P*_ref_ of the current slice with the original prediction result *P*_ori_ using a squeeze-and-excitation mechanism to derive the final prediction result *P*_fin_ for the current slice, as illustrated in Equation (6). In this context, *w*_ori_ and *w*_ref_ denote the weights associated with *P*_ori_ and *P*_ref_, respectively. A global pooling layer is employed to condense the comprehensive spatial information from both the original prediction *P*_ori_ and the refined result *P*_ref_ into a discrete feature vector as shown in Equation (7). We then enhance the channel dimension by a factor of *r* using two fully connected layers, followed by the application of a sigmoid activation function, which effectively captures the dependencies across different predictions, as demonstrated in Equation (8).

(6) $$P_{\text{fin}}=P_{\text{ori}}\cdot w_{\text{ori}}+P_{\text{ref}}\cdot w_{\text{ref}},$$

(7) $${global\_pool}^{\left(P\right)}=\frac{1}{H\times W}\sum_{i=1}^{H}\sum_{j=1}^{W}P(i,j),$$

(8) $$w=\sigma \;\left(fc2_{r}\left(fc1_{r}\left({\text{global}}_{\text{pool}(P)}\right)\right)\right),$$

### 2.3 | Joint loss for training

We train the network using a combination of cross-entropy loss and Dice loss, taking the axial plane as an example, as shown in Equations (9) and (10).

(9) $$L_{CE}(Y,P)=-\frac{1}{H\times W}\sum_{i=1}^{H}\sum_{j=1}^{W}[Y(i,j)log\;P(i,j)\;+(1-Y(i,j))log(1-P(i,j))],$$

(10) $$L_{DSC}\;(Y,P)=1-\frac{2\sum_{i=1}^{H}\sum_{j=1}^{W}Y(i,j)\cdot P(i,j)}{\sum_{i=1}^{H}\sum_{j=1}^{W}Y(i,j)+\sum_{i=1}^{H}\sum_{j=1}^{W}P(i,j)},$$

here, *P* represents the network’s prediction results, and (*i, j*) indicates the pixel’s position. In the network’s output, we obtain the original prediction result *P*_ori_ and the final prediction result *P*_fin_ for the current slice. The calculation of the axial plane loss is given in Equations (11) and (12), where *L*_ori_ is the loss for the original prediction result and *L*_fin_ is the loss for the final prediction result.

(11) $$L_{ori}=L_{DSC}\;\left(Y,P_{ori}\right)+L_{CE}\left(Y,P_{ori}\right),$$

(12) $$L_{fin}=L_{DSC}\;\left(Y,P_{fin}\right)+L_{CE}\left(Y,P_{fin}\right),$$

(13) $$L=\alpha \cdot L_{ori}+(1-\alpha )\cdot L_{fin},$$

The joint loss is denoted as *L*, with hyperparameters *α* used to balance the two components of the loss, as Equation (13) shows.

### 2.4 | Dataset details

This retrospective study was conducted following approval from the Institutional Review Board at our institution along with a Health Insurance Portability and Accountability Act (HIPAA) waiver, utilizing near-normal datasets previously obtained for clinical or research purposes. The participants in this study have a mean age of 50 years, with an age range of 21–79 years, and the study includes 34 men and 50 women. The dataset comprises non-contrast enhanced CT images with a slice spacing ranging from approximately 3 to 4 mm and an axial plane spatial resolution of 0.97 mm × 0.97 mm, which were acquired on either Philips or Siemens scanners. Each anatomical region, in particular, the axillary region and the lower cervical region, was annotated meticulously under supervision of a board-certified radiologist. This private dataset includes a total of 100 patient studies obtained from a single medical center, with 50 allocated for training, 10 for validation, and 40 for testing purposes. All images were converted to float32 format and *Z*-score normalized, utilizing predefined parameters: the mean intensity of 955.29 and a standard deviation of 222.93. The axillary region and the lower cervical region each comprises of two relatively symmetric, connected areas located on the left and right sides of the body as depicted in Figure 1.

### 2.5 | Implementation details

We implemented a slice adjacency-guided ensemble framework based on the PyTorch framework for open region segmentation. The weights for combining results from different directions, *w*_t_, *w*_s_, and *w*_c_, were set at 0.4, 0.2, and 0.4, respectively, based on empirical tuning through practical experience. The number of adjacent slices *l* is set to 1, and the number of previous and next *s* slice required for the current slice is also configured to 1 based on both prior work and the characteristics of our task. The correlation matching size *k* is set to 1, resulting in a 3 × 3 local window, as suggested in prior works (Dosovitskiy et al., 2015) as well as the ablation experiments shown in section 3.2.2, while the squeeze- and-excitation parameter *r* is empirically established at 64. Furthermore, the hyperparameter of the balanced loss, *α* is set to 0.5. All experiments were conducted using a single NVIDIA GeForce RTX 3090 GPU. For optimization, we employed the Stochastic Gradient Descent (SGD) algorithm with a batch size of 16, a learning rate of 0.01, a momentum of 0.9, and a weight decay of 0.001 to manage the network’s learning process. The training was executed over a total of 300 epochs.

### 2.6 | Evaluation metrics

The evaluation of segmentation accuracy involves several metrics, including the Dice similarity coefficient (DSC), the average symmetric surface distance (ASD), and the 95% Hausdorff Distance (HD95). These metrics are calculated in three-dimensional space. DSC is the primary metric used to assess how well the predicted segmentation overlaps with the ground truth labels, measuring the volumetric overlap and mismatch between them. ASD measures the average distance between the predicted surface and the ground truth surface. Similarly, HD95 focuses on local inconsistencies by identifying the 95th percentile of the Hausdorff distances calculated across all points. Improvements in segmentation performance are indicated by smaller values of ASD and HD95, as well as larger values of DSC.

## 3 | RESULTS

Initially, we compared the performance of our approach to that of the baseline model across various parameter configurations, as presented in Table 2. The results demonstrate that our method not only outperforms the baseline model with an equivalent number of parameters but also achieves optimal outcomes while utilizing a lower parameter count.

According to Table 2, our method demonstrates a 4.21% improvement in segmentation of the axillary region compared to the baseline method, while in the lower cervical region, the improvement is 6.85%. Figure 6 clearly shows that our approach places a greater emphasis on accurately handling edge details than the baseline model. Additionally, at lower number of parameters, the use of adjacent slices as input channels adversely affects segmentation performance in the lower cervical region. This suggests that, with limited parameters, introducing multiple channels can actually degrade performance due to the model’s constrained capacity to learn from small regions. However, with the same parameter count, our pixel displacement guidance strategy effectively alleviates this issue, substantially boosting the model’s segmentation performance. Figure 6 also displays the visual results for the baseline model at varying parameter counts, alongside those of our method.

Table 3 presents a comparative analysis between the proposed pixel displacement strategy and optical flow-based method. In the axillary region, our strategy improves boundary localization, reducing the HD95 distance from 8.01 to 7.10 mm and ASD from 1.69 to 1.62 mm. In the lower cervical region, the superiority becomes more pronounced, indicating enhanced capability in capturing step-like regions while maintaining comparable inference times.

Table 4 displays DSC values for our method for different slice orientations and also for the multi-view strategy. The findings presented in Table 4 reveal that the application of the multi-view ensemble approach resulted in a 1.27% improvement in the axillary region when compared to the axial plane results. In the lower cervical region, the improvement was greater, at 1.87%.

When assessing these regions against the planes that provided the least information, the axillary region exhibited a 3.35% improvement, while the lower cervical region demonstrated a 6.79% improvement. Figures 7 and 8 display representative segmentation outcomes using axial, sagittal, and coronal slices in the axillary region and cervical region, respectively. For example, the fourth column displays the results from training with axial slices, displayed across the axial, sagittal, and coronal planes. The final column illustrates the results achieved through the multi-view approach. By integrating the learning outcomes from different planes, we can see that the model’s segmentation accuracy in enhanced especially at open areas.

### 3.1 | Comparative experiments

In our comparative experiments, we assessed several leading medical image segmentation networks, including UNet^21^ (2D), UNet++^22^ (2D), 3D UNet^29^ (3D), EfficientSegNet^37^ (3D), SwinUNetR^56^ (3D), CSANet^57^ (2.5D), CSMA^58^ (2.5D) and CAT-Net^59^ (2.5D). The results from these comparisons clearly indicate that our model outperforms the other methods in terms of Dice coefficient, while also achieving competitive results in HD95 and ASD. The outcomes marked in bold represent the highest performance, while those highlighted with an underline signify the second-best performance. Since we find that the results achieved from the comparative methods do not satisfy the normality assumption, the Wilcoxon test was uniformly adopted to ensure analytical robustness. The results shown in Tables 5 and 6 underwent pairwise comparisons with FDR correction for *p*-values, showing a significant difference compared to ours across three metrics.

As illustrated in Table 5, UNet and UNet++ are 2D methods that face challenges in capturing 3D spatial information, resulting in relatively lower segmentation accuracy. Although both 3D-UNet and EfficientSegNet can process spatial data, they require more resources and demonstrate lower accuracy in comparison to our approach (as shown in Table S1). The latter three methods utilize 2.5D techniques, approximating 3D structures by analyzing 2D slices. While these enhance computational efficiency, they still fall short in accuracy and detail preservation when compared to fully 3D methods.

Our model not only achieves superior segmentation accuracy but also excels in HD95 and ASD metrics, highlighting its overall enhanced performance. We also observed that the version of our model that does not incorporate the multi-view strategy surpasses 3D methods, further underscoring its outstanding capabilities.

In the lower cervical region, our method delivers overall the best results in both Dice and ASD scores. The segmentation performance of the 3D model decreases in this area due to the step-like changes, as demonstrated in Table 6. The 3D model struggles to capture boundary details, particularly in regions with sharp transitions. In contrast, the 2.5D model is better suited to focus on local details and adapt to these sudden changes, resulting in improved segmentation accuracy and boundary clarity. Additionally, while pixel displacement enhances segmentation performance, it remains slightly less effective than attention-based networks, especially when addressing abrupt changes.

To evaluate the training efficiency and inference performance of the models, we conducted experiments. As shown in Table S1, the experiments compared different models across training time, GPU memory consumption during inference, and inference time. Figures 9 and 10 present representative segmentation results from the comparative experiments. From these figures, we observe that our method demonstrates high consistency and excellent segmentation accuracy, even without employing the multi-view ensemble strategy, thereby highlighting the advantages of the pixel displacement guidance approach. This guidance effectively captures dynamic changes and intricate boundary details, thereby enhancing the segmentation accuracy of open region boundaries and exhibiting remarkable performance in identifying both the starting and ending slices. Furthermore, the results shown in Figure 10 further validate the accuracy of our method in pinpointing the ending slice.

The implementation of the multi-channel ensemble strategy considerably improves the quality of segmentation results. This method not only addresses the segmentation challenges posed by step-like changes but also effectively reduces errors that may arise from relying on a single model. Consequently, the robustness of the segmentation results is enhanced.

### 3.2 | Ablation experiments

#### 3.2.1 | Impact of strategy selection on performance

To gain a deeper understanding of the contributions of each module within the system, we conducted ablation experiments that included the following in different combinations: (1) use of adjacent slices as supplementary input channels for the network; (2) utilization of pixel displacement information between adjacent slices to guide network segmentation in single-channel scenarios; (3) utilization of pixel displacement information between adjacent slices to guide network segmentation in three-channel scenarios; and (4) integration of results from multiple directions. When any of these strategies are omitted, our proposed framework reverts to a basic U-Net architecture. The results of these experiments are presented in Tables 7 and 8.

When we initially trained the model using only adjacent slices as input channels, the results revealed a slight improvement in performance for the axillary region, while the lower cervical region showed detrimental effects. This disparity was largely due to the region’s small size and the pronounced variations in the target area between slices. These findings demonstrate that relying solely on information from adjacent slices is insufficient to accurately capture the complex variations present in areas that are both small and highly variable. The pixel displacement strategy improves continuity between slices and aids in better tracking of dynamic changes in the target region. The experimental results indicated a notable improvement in the model’s performance, with segmentation accuracy increasing by 2.99% in the axillary region and by 8.49% in the lower cervical region.

The pixel displacement guidance strategy influences the model’s performance, particularly in multi-channel input scenarios. As demonstrated in Figure 11, integrating this strategy enhances performance whether a single-channel or multi-channel approach is used. Specifically, it optimizes feature extraction and context modeling by incorporating motion information, thereby improving the model’s accuracy. In multi-channel settings, the additional channels provide richer contextual information, leading to a more pronounced enhancement in performance. This, in turn, strengthens the model’s ability to capture temporal dependencies and motion cues effectively.

As demonstrated in Figure 12, to tackle the challenges posed by abrupt shape transitions, we introduce a multi-view ensemble strategy that extracts features at various scales from multiple perspectives, enabling us to manage abrupt shape variations and minimize errors that may propagate to subsequent slices. Figure S2 provides a comparative visualization of the initial error cases and their improved counterparts after applying the multi-view integration strategy. Table S3 shows the results on overlap slices, which further demonstrate the positive effects of multi-view strategy.

#### 3.2.2 | Impact of configuration on model performance

Our study investigates the mechanistic impacts of core parameter configurations (*k, md, l*) in the pixel displacement strategy on medical image segmentation performance through multi-level ablation experiments as shown in Table S4.

Additionally, we conducted a weighted fusion of outcomes from three directions. Different weights were assigned to each direction to explore their contributions to the final results. Figure S3 illustrates the impact of different weight allocation strategies on the final fusion outcomes.

## 4 | DISCUSSION

In the datasets for the axillary and lower cervical regions, our method has demonstrated a considerable advantage. As shown in Table 2, our model achieves a relatively high Dice score even with a lower number of parameters. However, when the model’s learning capacity is insufficient, especially in small regions with abrupt changes like the lower cervical region, using adjacent slices as input channels to gather contextual information may have a negative impact. In such cases, utilizing pixel displacement information can greatly mitigate the negative effects caused by using adjacent slices as input channels and enhance the model’s segmentation performance for these areas, as illustrated by Figures 6, 9, and the blue curves in Figure 11, specifically in parts b and d.

Nevertheless, due to abrupt changes in the region, pixel displacement information may contain errors. To address this, we introduced a multi-view ensemble strategy that integrates different content learned from various perspectives, effectively alleviating the issue of abrupt changes and limiting the upper and lower boundary errors of the open regions, as shown in Table 4 and Figures 7 and 8.

Furthermore, as indicated in Tables 5 and 6, 2D methods struggle to capture the spatial relationships between slices, leading to relatively low Dice coefficients, HD95, and ASD metrics. In contrast, 3D methods and 2.5D methods that utilize spatial information show a marked improvement in performance compared to 2D methods, reflecting the importance of spatial information for segmentation accuracy.

Although 3D models can process spatial information, they require more resources, and for the EfficientSegNet model, the segmentation results are not satisfactory, likely due to its use of anisotropic convolution and the reduction of channel numbers before sampling in the decoder part, which is not suitable for our two regions, especially the small mutation areas in the lower cervical region. In addition, our compared 2.5D methods, while improving computational efficiency, still fall short in terms of accuracy and detail preservation compared to full 3D methods. Our method not only achieves higher segmentation accuracy but also excels in HD95 and ASD metrics, highlighting an overall enhancement in performance. Particularly in the axillary region, we observed that our version without the integration strategy performs better than 3D methods, further highlighting its exceptional capability. However, when dealing with abrupt changes, the version without the integration strategy is still slightly inferior to attention-based networks.

We conducted paired *t*-tests for the three metrics across both regions, and the results indicate a statistically significant difference between our method and other approaches. Figures 9 and 10 display representative segmentation results from the comparative experiments. From these figures, we observe that our method exhibits high consistency and superior segmentation accuracy, even without employing the multi-view integration strategy, demonstrating the advantage of the pixel displacement guidance method in capturing dynamic changes and intricate boundary details, effectively addressing the segmentation challenges of open regions.

## 5 | CONCLUSION

This study proposes a 2.5D segmentation framework guided by pixel displacement. The framework improves segmentation performance in open regions by using pixel displacement information and reduces boundary errors caused by abrupt changes through a multi-view ensemble strategy. Experiments on two in-house datasets show that our approach outperforms existing 2D, 3D, and 2.5D methods. Even without the multi-view integration strategy, our method achieves competitive results, especially in the axillary region, where it surpasses traditional 3D methods. After incorporating the multi-view ensemble strategy, our method further exceeds state-of-the-art methods by 1.30% and 2.07% in Dice coefficients, demonstrating superior segmentation accuracy and robustness.

## Supplementary Material

NTCSR8

Additional supporting information can be found online in the Supporting Information section at the end of this article.

## Figures and Tables

**FIGURE 1:**
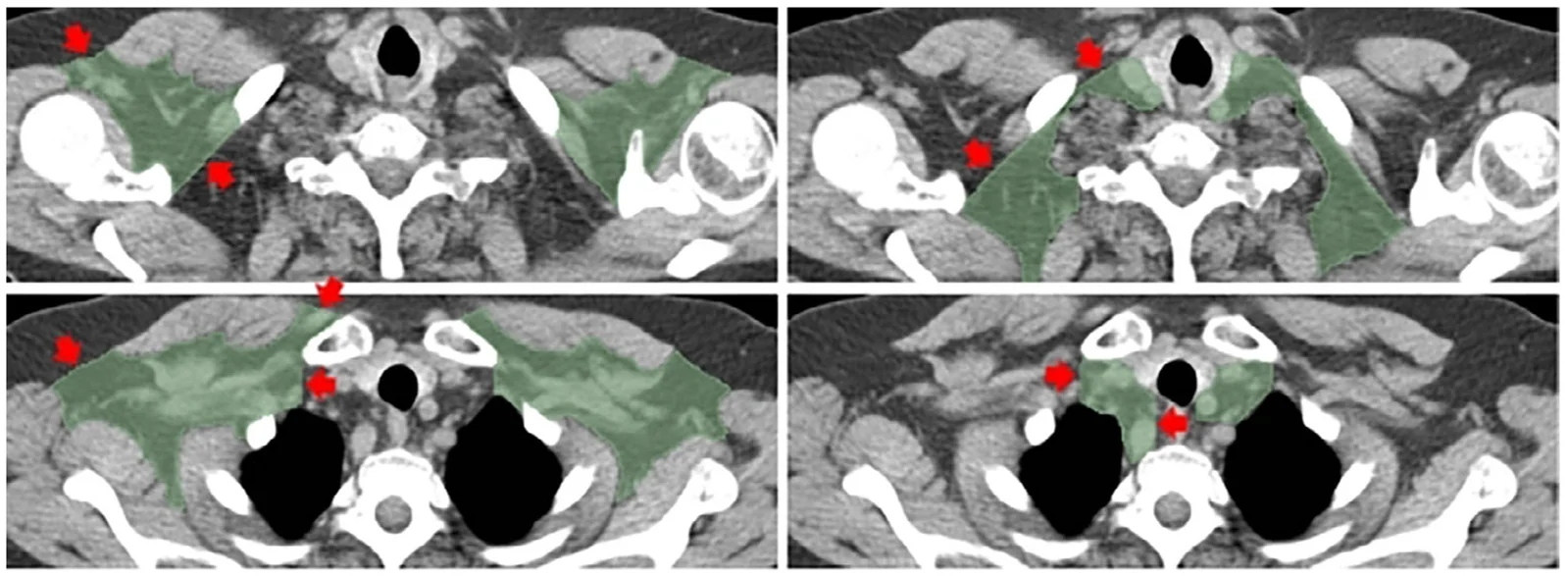
The openness of the axillary and lower cervical regions. The first column shows the axillary region, and the second column shows the lower cervical region. Red arrows point to portions of open regions without observable intensity boundaries. As these regions are symmetrical, only their right sides are labeled with arrows.

**FIGURE 2:**
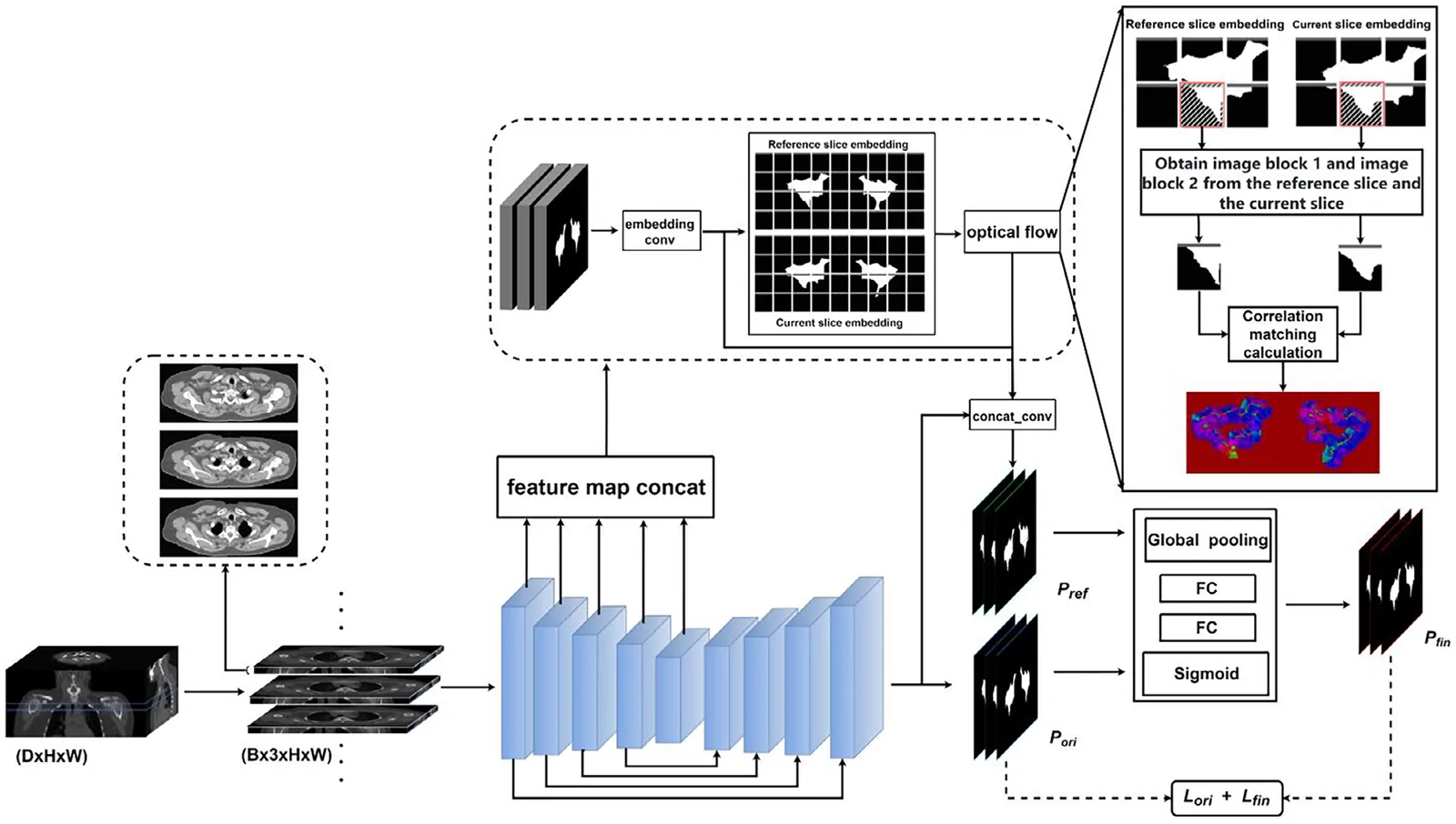
A visual representation of our workflow for open region segmentation, improving segmentation performance by integrating multi-slice and multi-view information.

**FIGURE 3:**
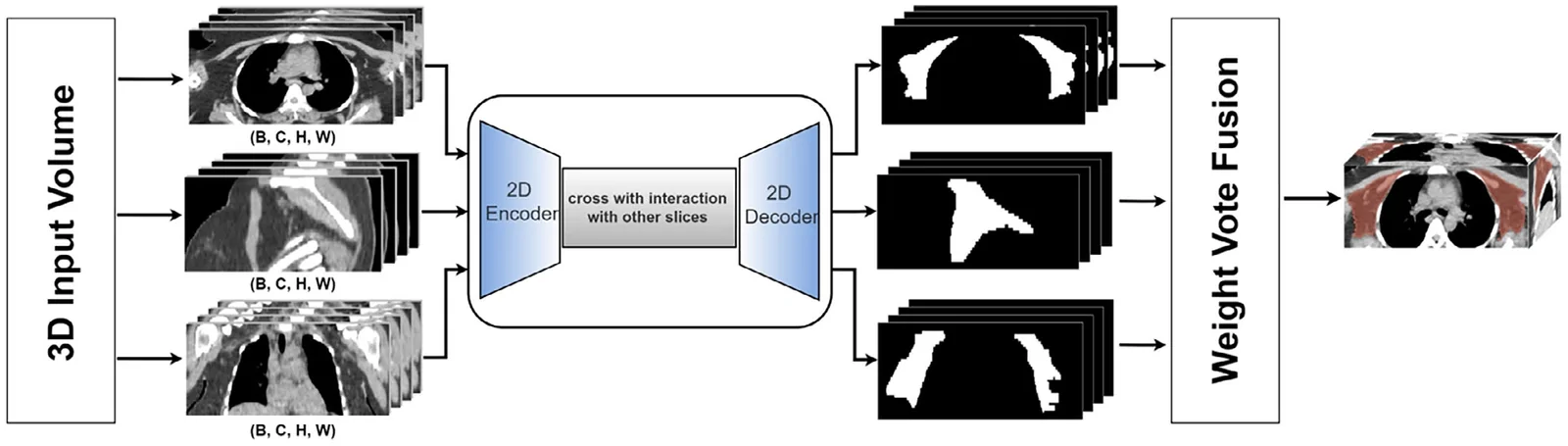
Data processing approach using multiple planes in the proposed method. Re-slice the same input data along the axial, sagittal, and coronal planes, stack consecutive slices in each direction, and then process them through the same network. Finally, combine the outputs of the individual networks using weight vote strategy. B and C represent the batch size and the input slice number, respectively.

**FIGURE 4:**
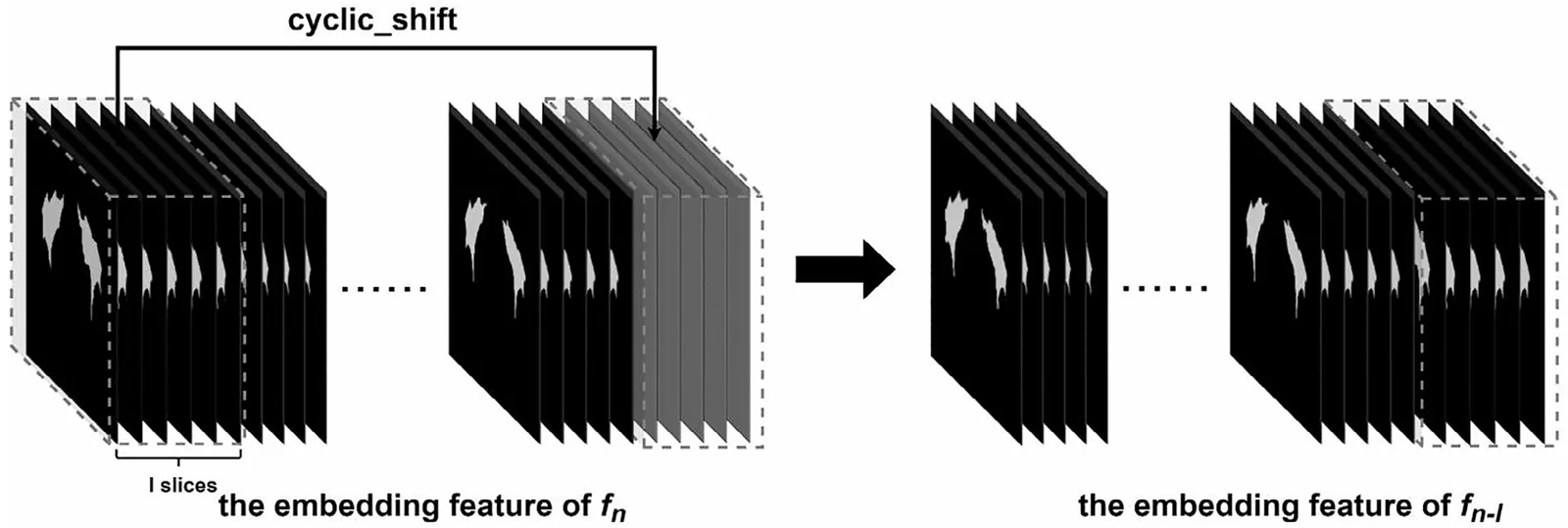
Cyclic shift of the embedding features of the current slice to obtain the embedding features of the adjacent slices.

**FIGURE 5:**
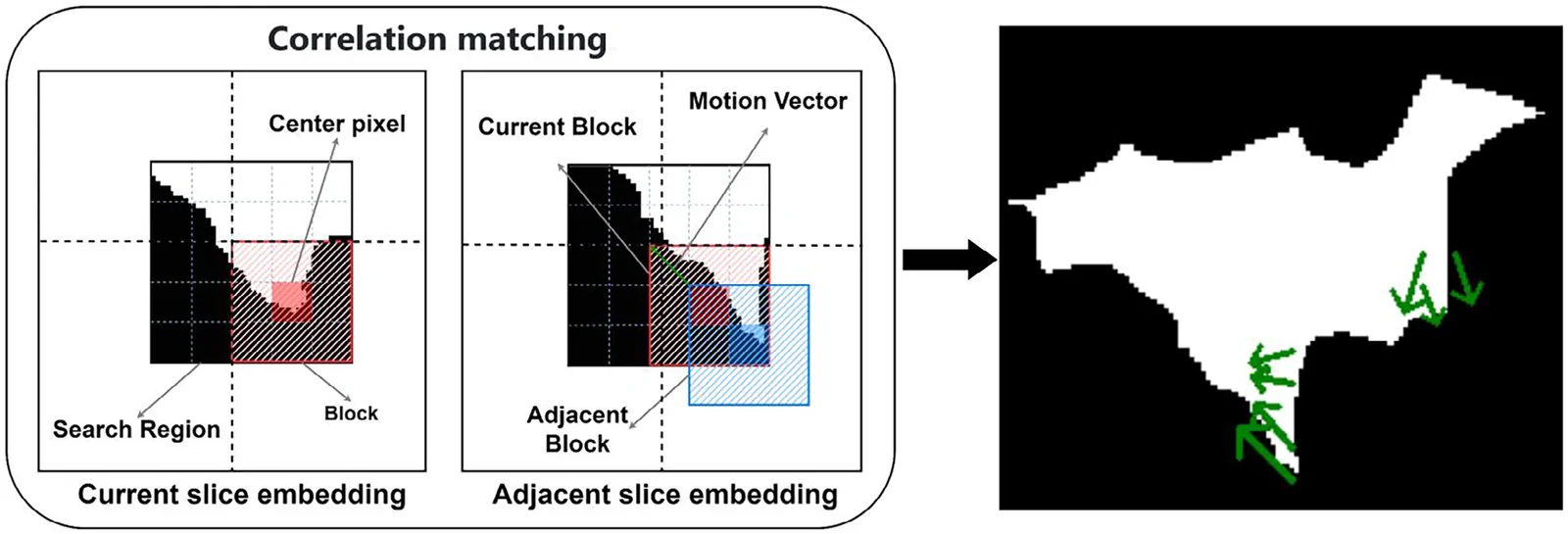
Illustrate of the correlation matching procedures. The movement of pixel *x* is represented by calculating the degree of matching between the embedding features of adjacent and current slice. By calculating the displacement of all pixels within the search region. The distance map shown on the right side of the figure can be obtained, where the direction of the arrow represents the direction of pixel movement, and the length of the arrows represent the movement distance. The arrows on the right of the figure are examples.

**FIGURE 6:**
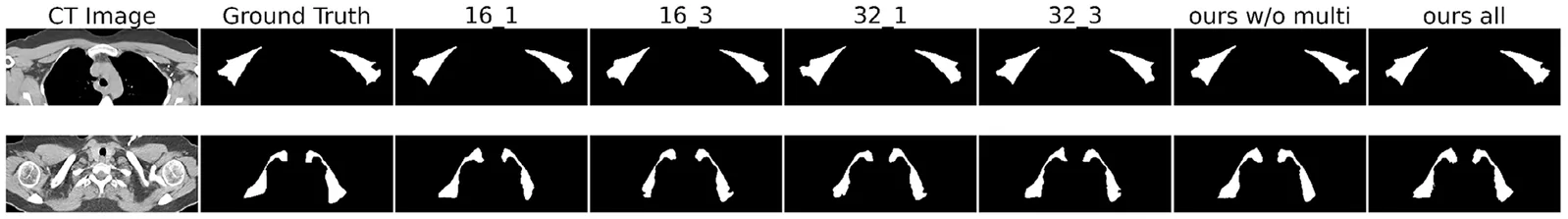
A comparison of experimental results between a simple U-Net model and an enhanced U-Net model on the axillary region and the lower cervical region, evaluated under different parameter counts (note that the enhanced model incorporates adjacent slices as additional channel inputs and pixel displacement information guidance strategies). For instance, the notation 16_1 denotes the output of the first convolutional layer, where the input, a single-channel image, is transformed into a feature map with 16 channels.

**FIGURE 7:**
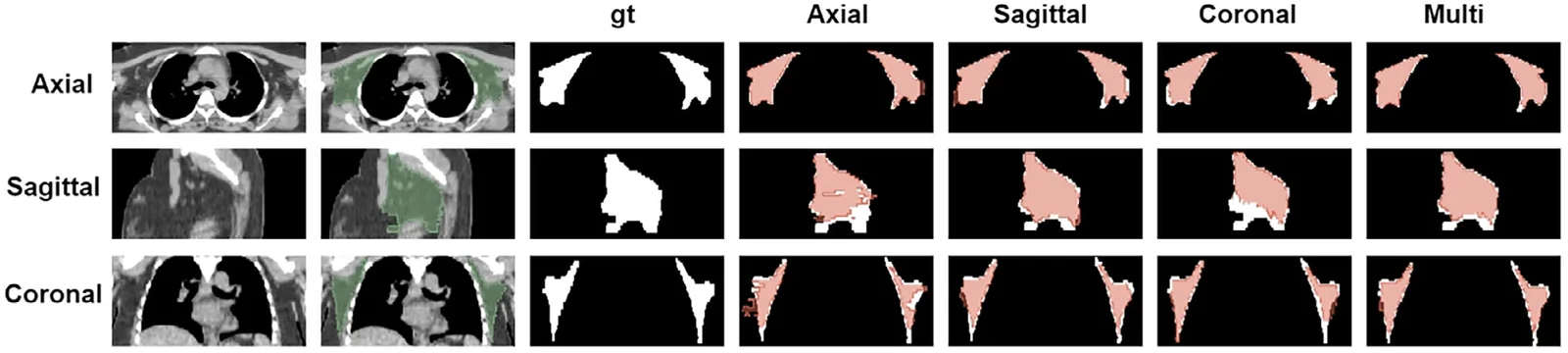
Segmentation results obtained for the axillary region with individual views and the multi-view approach. The results are displayed along the axial plane, coronal plane, and sagittal plane.

**FIGURE 8:**
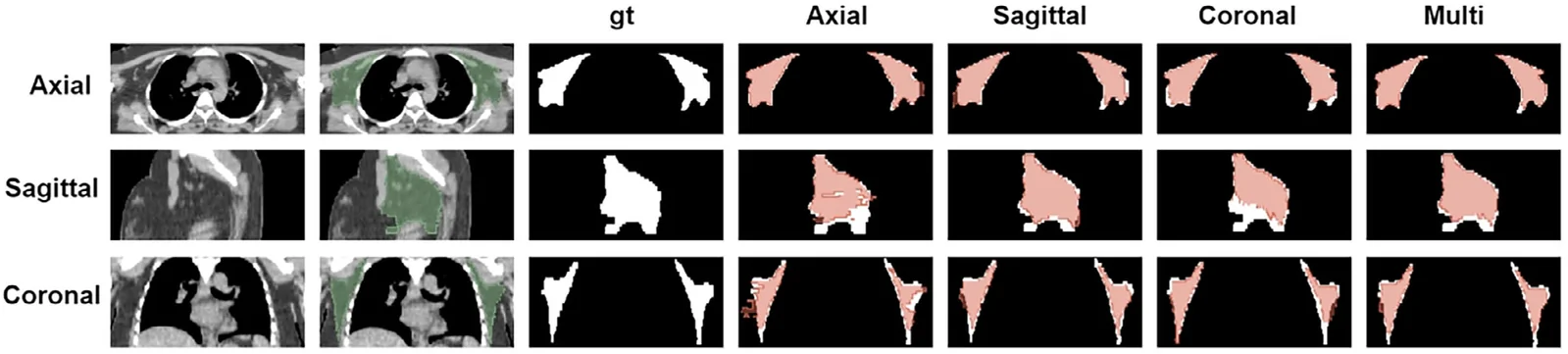
Segmentation results obtained for the lower cervical region with individual views and the multi-view approach. The results are displayed along the axial plane, coronal plane, and sagittal plane.

**FIGURE 9:**
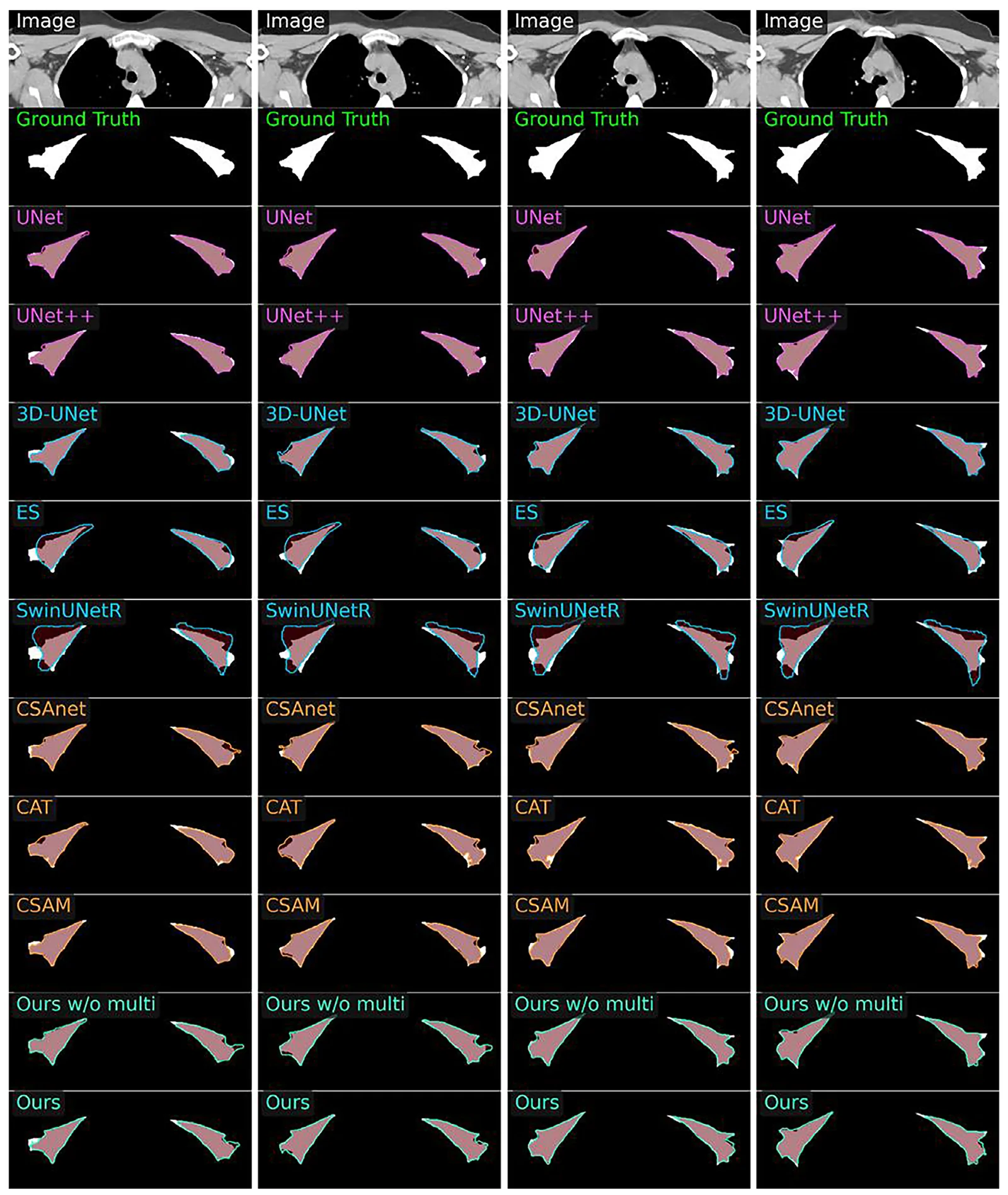
Comparison of representative segmentation results of our model with current mainstream medical image segmentation networks for the axillary region.

**FIGURE 10:**
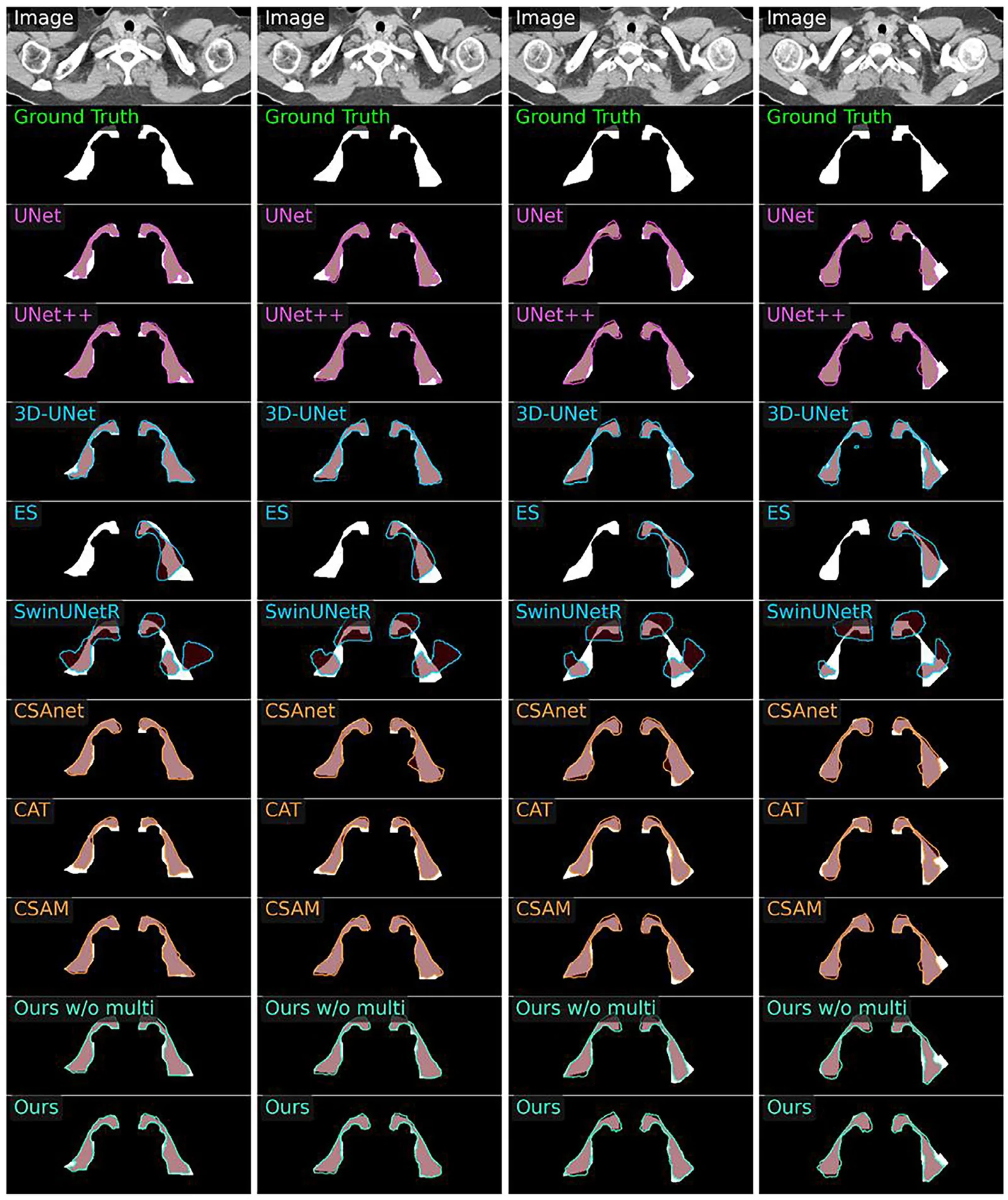
Comparison of representative segmentation results of our model with current mainstream medical image segmentation networks for the lower cervical region.

**FIGURE 11:**
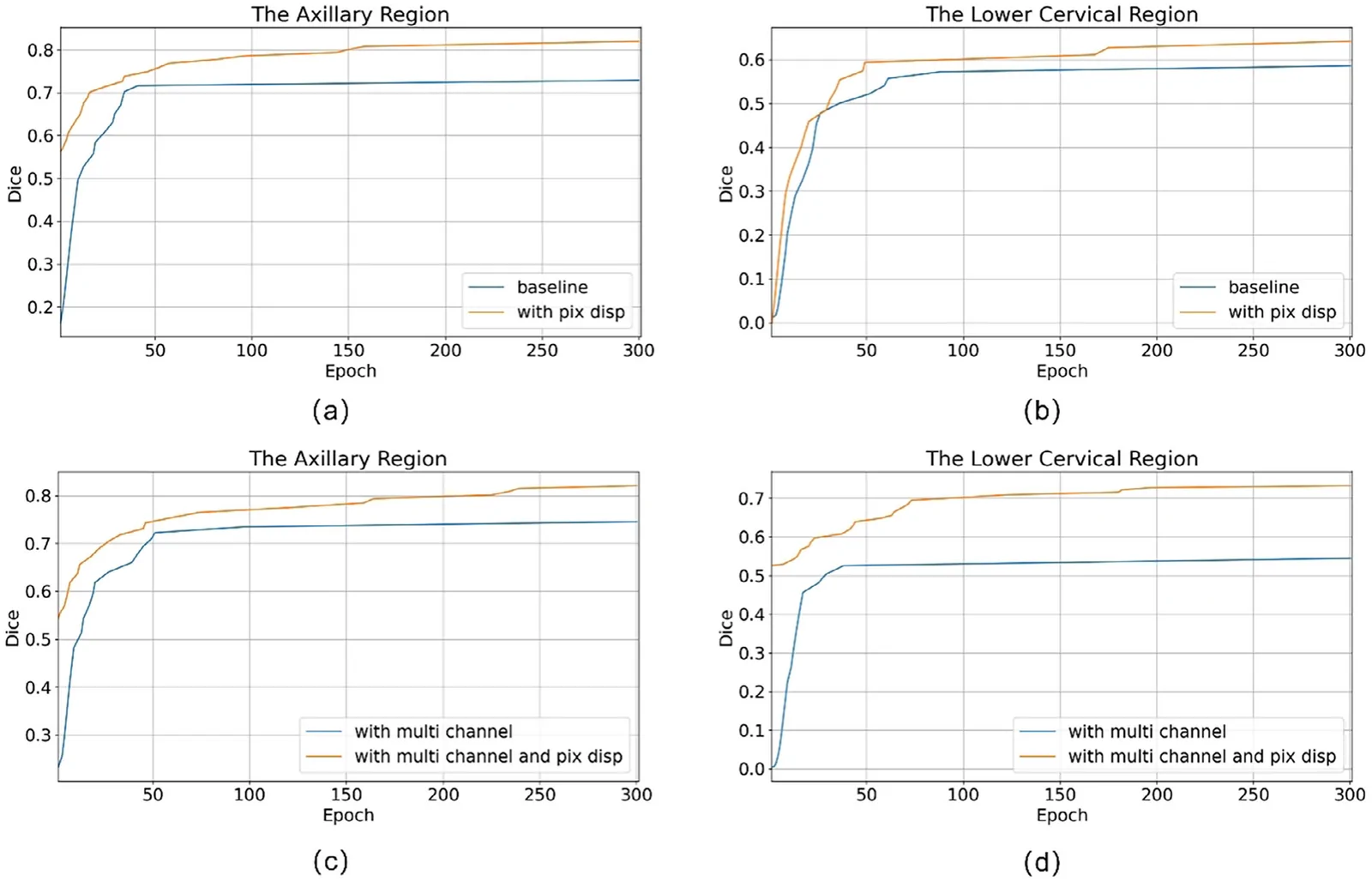
The impact of adding the multi-channel strategy and pixel tracking strategy on model training. (a) and (b) introduce pixel displacement information based on the baseline, while (c) and (d) incorporate pixel displacement information on a multi-channel basis. The original data curve has been smoothed using the Exponential Moving Average (EMA). The smoothed curves are calculated using an EMA with a smoothing factor α of 0.1.

**FIGURE 12:**
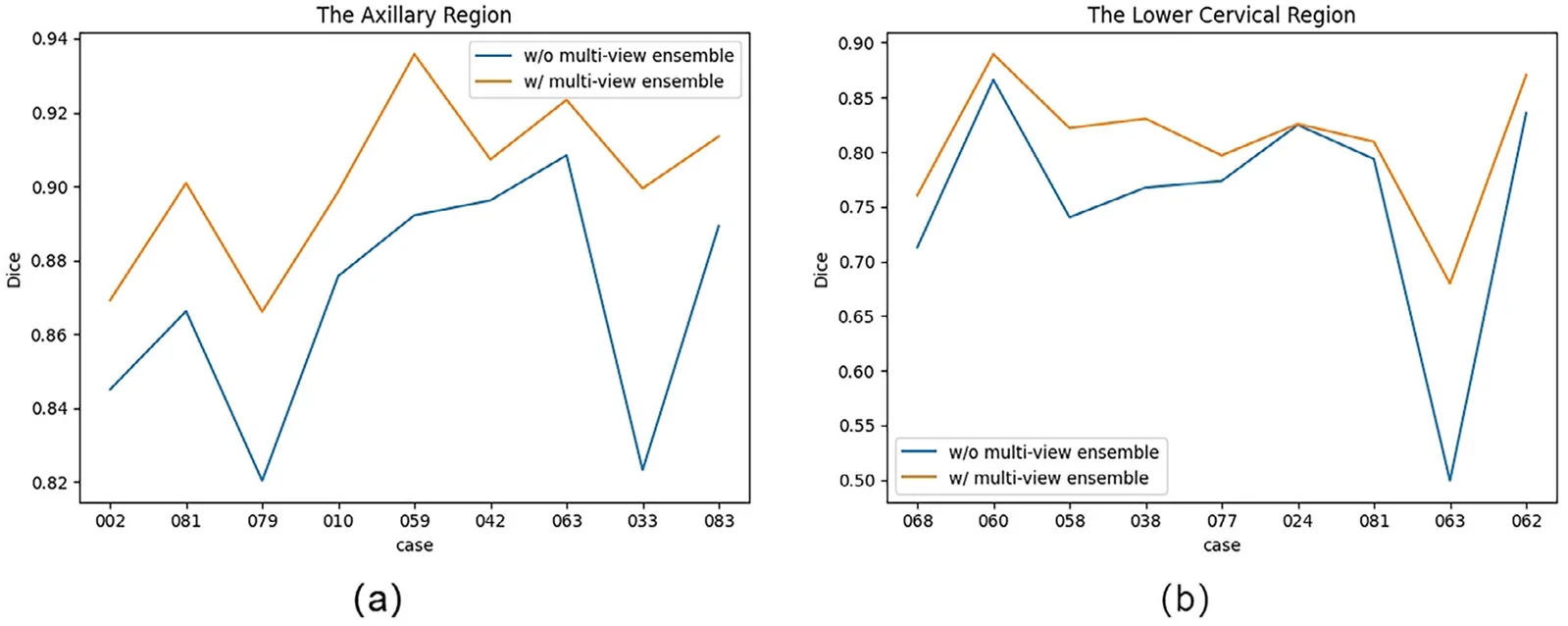
The data randomly selected from the test set are shown: (a) axillary region, and (b) lower cervical region. The *x*-axis represents the names of the cases selected.

**TABLE 1:** Descriptions of the axillary and lower cervical regions.

Region	Description
Axillary region	The region encompassing the left and right sides of the upper thorax surrounded by portions of thoracic and upper extremity skeletal musculature that contain axillary lymph nodes, neurovascular structures, and adipose tissue. This region extends from the superior slice through the pectoralis minor muscle insertions into the scapulae to the inferior slice through the pectoralis major muscles origins from the rib cage. In-plane boundaries utilize contours along surrounding structures, including skeletal muscles, fascial planes, and osseous structures, that vary from slice to slice.
Lower cervical region	The region encompassing the left and right sides of the lower neck surrounded by portions of neck, thorax, and upper extremity skeletal musculature that contain lower cervical and supraclavicular lymph nodes, neurovascular structures, and adipose tissue. This region extends from the superior slice located 15 mm above the lung apices to the inferior slice through the upper aspect of the manubrium. In-plane boundaries utilize contours along surrounding structures, including skeletal muscles, visceral structures, and osseous structures, that vary from slice to slice.

**TABLE 2:** Dice similarity coefficient (DSC) for our method (without the multi-view ensemble strategy) compared with those of U-Net structures with various parameter settings.

Method	Number of parameters	Channel		Axillary region	Lower cervical region
		1	3		
U-Net (baseline)	14.28 M	√	–	0.8477 ± 0.0834	0.7171 ± 0.0709
		–	√	0.8599 ± 0.0666	0.7007 ± 0.0701
	28.69 M	√	–	0.8719 ± 0.0367	0.7375 ± 0.0580
		–	√	0.8760 ± 0.0371	0.7448 ± 0.0540
Ours	14.28 M	√	–	0.8871 ± 0.0399	0.7631 ± 0.0675
		–	√	**0.8898 ± 0.0313**	**0.7856 ± 0.0521**

**TABLE 3:** Pixel displacement strategy vs. optical flow method: performance comparison.

Metrics	Region	Dice	HD95 (mm)	ASD (mm)	Infer time (s)
Flow	AxR	0.8862 ± 0.0320	8.01 ± 3.79	1.69 ± 0.93	3.07 ± 0.45
Pixel		0.8898 ± 0.0313	7.10 ± 2.66	1.62 ± 0.63	3.18 ± 0.55
Flow	LCR	0.7714 ± 0.0713	10.77 ± 13.98	2.19 ± 2.85	1.75 ± 0.023
Pixel		0.7856 ± 0.0521	8.26 ± 10.12	2.30 ± 2.86	1.86 ± 0.45

**TABLE 4:** Dice similarity coefficient (DSC) for our method on the three anatomical planes individually and for the multi-view ensemble strategy.

View	Axillary region	Lower cervical region
Axial	0.8898 ± 0.0313	0.7856 ± 0.0521
Sagittal	0.8776 ± 0.0301	0.7711 ± 0.0606
Coronal	0.8690 ± 0.0363	0.7364 ± 0.0608
Multi-view	**0.9025 ± 0.0255**	**0.8043 ± 0.0457**

**TABLE 5:** Comparison of segmentation results for the axillary region.

Method	Dice	HD95 (mm)	ASD (mm)
UNet	0.8477 ± 0.0834	12.26 ± 8.32	1.90 ± 1.03
UNet++	0.8583 ± 0.0517	10.25 ± 6.45	1.71 ± 0.74
3D UNet	0.8893 ± 0.0370	8.10 ± 3.55	1.73 ± 0.92
EfficientSegNet	0.7054 ± 0.0651	17.73 ± 60.43	3.24 ± 0.52
SwinUNetR	0.7884 ± 0.0478	9.42 ± 4.63	2.54 ± 0.48
CSANet	0.8832 ± 0.0379	9.93 ± 8.95	2.74 ± 1.82
CSMA	0.8883 ± 0.0425	9.64 ± 7.82	1.61 ± 1.31
CAT-Net	0.8520 ± 0.0434	10.21 ± 5.98	3.66 ± 3.41
**Ours**	**0.9025 ± 0.0255**	**5.38 ± 1.75**	**1.13 ± 0.44**

*Note*: All models show a significant difference compared to ours across three metrics (Wilcoxon signed-rank test, *p*-value < 0.05).

**TABLE 6:** Comparison of segmentation results for the lower cervical region.

Method	Dice	HD95 (mm)	ASD (mm)
UNet	0.7375 ± 0.0580	11.02 ± 11.77	2.46 ± 2.63
UNet++	0.7421 ± 0.0529	8.62 ± 8.06	2.25 ± 1.95
3D UNet	0.7365 ± 0.0658	8.49 ± 8.47	1.83 ± 0.66
EfficientSegNet	0.6089 ± 0.0741	10.36 ± 16.59	3.49 ± 1.61
SwinUNetR	0.6475 ± 0.0478	9.41 ± 8.38	3.28 ± 0.63
CSANet	0.7811 ± 0.0605	9.14 ± 10.65	2.21 ± 2.42
CSMA	0.7858 ± 0.0569	8.43 ± 11.86	2.40 ± 3.08
CAT-Net	0.6894 ± 0.0841	16.65 ± 14.36	5.61 ± 4.13
Ours	**0.8043 ± 0.0457**	**7.86 ± 11.63**	**1.26 ± 0.41**

*Note*: All models show a significant difference compared to ours across three metrics (Wilcoxon signed-rank test, *p*-value < 0.05).

**TABLE 7:** Summary of ablation study results for the axillary region showing the impact of each component of our method on the final performance to assess the importance of each part.

Multi-channel	Pixel displacement	Multi-view	Dice	HD95 (mm)	ASD (mm)
–	–	–	0.8477 ± 0.0834	12.26 ± 8.32	1.90 ± 1.03
√	–	–	0.8599 ± 0.0666	10.98 ± 6.89	1.64 ± 0.77
–	√	–	0.8871 ± 0.0399	8.27 ± 3.88	1.69 ± 0.87
√	√	–	0.8898 ± 0.0313	7.10 ± 2.66	1.62 ± 0.63
√	√	√	**0.9025 ± 0.0255**	**5.38 ± 1.75**	**1.13 ± 0.44**

**TABLE 8:** Summary of ablation study results for the lower cervical region showing the impact of each component of our method on the final performance to assess the importance of each part.

Multi-channel	Pixel displacement	Multi-view	Dice	HD95 (mm)	ASD (mm)
–	–	–	0.7171 ± 0.0709	11.52 ± 11.87	3.17 ± 3.14
√	–	–	0.7007 ± 0.0701	13.33 ± 14.51	2.78 ± 3.02
–	√	–	0.7631 ± 0.0675	9.25 ± 11.24	2.46 ± 2.84
√	√	–	0.7856 ± 0.0521	8.26 ± 10.12	2.30 ± 2.86
√	√	√	**0.8043 ± 0.0457**	**7.86 ± 11.63**	**1.26 ± 0.41**
