# Supplementary material for: Open area segmentation in CT images based on pixel displacement and multi-view with application in the axillary and lower cervical regions
Source: Med Phys. Author manuscript; Available in PMC 2026 Oct 29. (PMC13717279; doi:10.1002/mp.70210)
Supplement: Supplementary file 1 [file NIHMS2216491-supplement-NTCSR8.docx]

**TABLE S1 Pseudocode of the proposed method**

Table S1 Pseudocode of the proposed method

***Algorithm***

**Input:** Backbone features of current slice $f_{n}$

**Output:** Final segmentation of current slice $P_{n}^{fin}$

**Begin**

Step 1: Feature Extraction & Propagation

$f_{n}$← Backbone Concat	// Extract features

$f_{n}^{emb}$, $f_{n-l}^{emb}$← Embedding ($f_{n}$), Cyclic Shift ($f_{n}^{emb}$) // Propagate context

Step 2: Pixel Displacement Calculation

$D_{n}$← Correlation Matching ($f_{n}^{emb}$, $f_{n-l}^{emb}$) // Compute displacement field

Step 3: Feature Refinement

$P_{n-l}^{ref}$← Conv Block ($D_{n}$, $P_{n-l}^{ori}$, $f_{n}$) // Integrate guidance

Step 4: Attention-based Fusion

$P_{n}^{fin}$← SEFusion($P_{n}^{ref}$, $P_{n}^{ori}$)	// Generate final result

**End**

**TABLE S2 Training time vs. inference time vs. GPU memory usage during inference.**

Table S2 presents a comparison of different models in terms of training duration, GPU memory consumption during inference, and inference time. Our proposed method demonstrates a well-balanced performance across these metrics. Specifically, the method requires approximately 72 hours for training, occupies 5756 MB of GPU memory during operation, and achieves an inference time of 1.81 seconds. While the inference speed is marginally slower than 2D UNet, it substantially outperforms 3D approaches. This optimized configuration maintains relatively low memory usage while delivering competitive inference speed, coupled with moderate training requirements.

Table S2 Training time vs. inference time vs. GPU memory usage during inference.

| Method | Train Time (h) | Infer Time (s) | Memory (MB) |
| --- | --- | --- | --- |
| UNet | 24.6 | 0.76 | 2826 |
| UNet++ | 35.4 | 1.99 | 3379 |
| 3D UNet | 75.5 | 3.03 | 6068 |
| EfficientSegNet | 110.1 | 4.41 | 5884 |
| CSANet | 46.5 | 3.11 | 2388 |
| CSMA | 27 | 1.03 | 2839 |
| CAT-Net | 144 | 10.22 | 8120 |
| Ours (w/o multi-view) | 72 | 1.81 | 5756 |

**TABLE S3 Comparative HD95 Analysis Across Axial, Multi-View, and Overlap Slices Evaluations**

The Overlap Slices results demonstrate the model's true segmentation potential once frame number prediction bias is removed. The difference between multi-view fusion results and this potential value indicates areas for improvement in frame number positioning accuracy. The significant gap between axial single-view results and this potential confirms that frame number prediction deviations affect segmentation quality.

In the AxR, the HD95 metric improved from 7.10 to 5.38 with multi-view fusion, effectively integrating angular information. The HD95 value from the overlapping slice set in this area is 5.29, closely matching the multi-view fusion result, suggesting comparable segmentation performance to ideal frame positioning.

In the LCR, the axial single-view HD95 is 8.26 mm. After multi-view fusion, the HD95 improves to 7.86 mm. The slight increase in standard deviation may be due to multi-view fusion being more sensitive to complex anatomical structures, leading to performance variability across cases.

Table S3 Comparative HD95 Analysis Across Axial, Multi-View, and Overlap Slices Evaluations

| Region | Axial (mm) | Multi-view (mm) | Overlap-region (mm) |
| --- | --- | --- | --- |
| AxR | 7.10 ± 2.66 | 5.38±1.75 | 5.29 ± 2.04 |
| LCR | 8.26 ± 10.12 | 7.86±11.63 | 4.56 ± 2.49 |

Note: Axial and Multi-view HD95 values are computed between the ground truth (GT) and prediction across the entire case volume, whereas Overlap Slices HD95 is calculated exclusively on the overlapping slices where both GT and prediction are present.

**TABLE S4 Results of the model in the axillary and lower cervical regions under different parameter configurations**

As shown in Table S4, quantitative evaluations were conducted in the axillary region and lower cervical region, respectively. Specifically, $k$ controls the neighborhood range for correlation matching, $md$ denotes the maximum displacement radius defining spatial constraints, $l$ specifies the hierarchical propagation level across adjacent slices.

In the axillary region achieves the highest Dice score and minimal boundary error with the (2, 3, 1) configuration, demonstrating that moderately enlarging the matching radius enhances local structural consistency. Notably, parameter $l$ exhibits a nonlinear impact. When $k=1$ in the axillary region, increasing $l$ from 1 to 3 worsens HD95 and increases ASD variance, suggesting excessive slice propagation can lead to error accumulation.

Table S4 Results of the model in the axillary and lower cervical regions under different parameter configurations.

| (*k*, *md*, *l*) | AxR | | |
| --- | --- | --- | --- |
|  | Dice | HD95 (mm) | ASD (mm) |
| (1, 3, 1) | 0.8898±0.0313 | 7.10±2.66 | 1.62±0.63 |
| (1, 3, 2) | 0.8837±0.0388 | 7.98±3.92 | 1.93±1.06 |
| (1, 3, 3) | 0.8896±0.0361 | 7.56±4.14 | 1.76±1.12 |
| (2, 3, 1) | 0.8907±0.0358 | 7.06±3.80 | 1.61±0.72 |
| (2, 3, 2) | 0.8845±0.0394 | 7.99±3.88 | 1.91±1.05 |
| (2, 3, 3) | 0.8895±0.0365 | 7.57±4.10 | 1.76±1.12 |
|  | LCR | | |
| (1, 3, 1) | 0.7856±0.0521 | 8.26±10.12 | 2.30±2.86 |
| (1, 3, 2) | 0.7884±0.0681 | 8.76±11.30 | 2.35±2.86 |
| (1, 3, 3) | 0.7904±0.0628 | 8.24±10.49 | 2.32±2.69 |
| (2, 3, 1) | 0.7788±0.0646 | 8.84±11.12 | 2.21±2.74 |
| (2, 3, 2) | 0.7912±0.0616 | 8.57±11.34 | 2.38±2.93 |
| (2, 3, 3) | 0.7788±0.0676 | 8.54±10.89 | 2.19±2.84 |

In contrast, in the lower cervical region, with $k=1$, while increasing $l$ slightly raises the Dice score, it also increases HD95 and ASD fluctuations. This indicates that $l$ selection differently affects model performance across anatomical regions. Specifically, a larger $l$ in the lower cervical region can enhance the Dice score but also results in greater HD95 and ASD fluctuations, implying excessive slice propagation here also introduces errors and affects stability.

Further analysis reveals that setting $k=1$ and $l=1$ offers the best overall model performance in both regions. In the axillary region, this configuration nearly maximizes the Dice score while maintaining low HD95 and ASD values. In the lower cervical region, although the Dice score is slightly lower than with higher $l$ values, the smaller HD95 and ASD fluctuations indicate higher stability, which is crucial for clinical reliability.

Compared to configurations with larger $k$ values, which can reduce performance fluctuations caused by different $l$ values in the axillary region, they lead to increased HD95 and significantly reduced stability in the lower cervical region. This means that while a larger $k$ can improve the Dice score in the lower cervical region, it does so at the cost of boundary precision and stability.

In summary, selecting $k=1$ and $l=1$ in both regions is a globally optimal solution for system robustness. In the axillary region, it maintains high performance without significant error accumulation from $l$. In the lower cervical region, it sacrifices Dice score slightly for substantially better stability and boundary precision, achieving a performance balance across different anatomical regions and enhancing the model's overall effectiveness and reliability.
